# Molecular Signatures in Urologic Tumors

**DOI:** 10.3390/ijms140918421

**Published:** 2013-09-06

**Authors:** Spencer Larkin, Natasha Kyprianou

**Affiliations:** 1 Departments of Urology, University of Kentucky College of Medicine, Lexington, Kentucky, KY 40536, USA; E-Mail: spencer.larkin@uky.edu; 2 Department of Molecular and Cellular Biochemistry, University of Kentucky College of Medicine, Lexington, Kentucky, KY 40536, USA; 3 Department of Toxicology, University of Kentucky College of Medicine, Lexington, Kentucky, KY 40536, USA; 4 Department of Pathology, University of Kentucky College of Medicine, Lexington, Kentucky, KY 40536, USA

**Keywords:** miRNAs, vascularity, therapeutic resistance, circulating tumor cells, prostate cancer, bladder cancer, renal cancer

## Abstract

Urologic tumors continue to represent a huge fraction of cancer cases in the United States, with over 376,310 estimated new diagnoses in 2013. As with many types of tumors, urologic tumors vary greatly in their phenotype, ranging from minimally invasive to malignancies possessing great metastatic potential. The increasing need for more efficient and less invasive methods of cancer detection, as well as the ability to predict severity of the disease phenotype is readily evident—yet reliable methods remain elusive in a clinical setting today. Comprehensive panels of gene clusters are being developed toward the generation of molecular signatures in order to better diagnose urologic malignancies, and identify effective treatment strategies in the emerging era of personalized medicine. In this review, we discuss the current literature on the credibility and biomarker value of such molecular signatures in the context of clinical significance relating to the pathological aggressiveness of urologic tumors (prostate, bladder and renal cancer)—also exploiting their predictive potential in the response to treatment.

## 1. Introduction

In 2013, an estimated 58,610 deaths in the United States will result from prostate, bladder and kidney cancers [1]. Despite a tremendous amount of research effort, reliable and non-invasive methods for detecting malignancy and projecting disease course remain elusive in a clinical setting. Absence of a singular marker of malignancy calls for a shift toward examining more comprehensive genetic profiles of tumors, which has the enormous potential to provide treatment approaches tailored to the individual. These molecular signatures, or unique genetic features distinguishing tumor cells from normal tissue, have generated tremendous interest as a potential means to reliably identify disease and progression.

The use of molecular signatures to detect and determine rate of progression in various cancers represents the latest shift in moving toward identifying initial events contributing to early stage tumors, ultimately allowing for optimizing therapeutic response and treatment outcomes. Indeed the need for biomarkers with high predictive value in tumor progression to advanced disease and requiring less invasive procedures (than characterization of tumor morphology) is now higher than ever for urologic malignancies. Since the 1960’s, scoring of urologic tumors, such as the Gleason Score of prostatic tumors based largely on morphological characteristics, has been common practice. “Primitive” as the pursuit of morphological differences might seem, and with cell morphology alone proven to be an imprecise predictor of cancer progression in the past, one must recognize that factors controlling tissue architecture, cell polarity and signaling processes such as epithelial-mesenchymal-transition (EMT), invasion and cell adherence may represent attractive options within a signature collection. EMT is now viewed as an essential early step in cancer metastasis. Significantly the switching between cell phenotypes that occurs during EMT is specified by certain microRNAs, (small non-coding RNAs regulating gene expression post-transcriptionally), thus enabling expansion of the molecular platforms for the pursuit of molecular signatures, towards predicting the metastatic course of cancer progression, as well as therapeutic response. In this review, we discuss the increasingly recognized value of biomarkers in molecular signatures and their impact on identification of the three major types of urologic tumors—with a focus on projecting their clinical impact in patient diagnosis and treatment.

## 2. Prostate Cancer

Prostate cancer remains the second most common cancer-related death in men, and will result in more than 29,720 estimated deaths in 2013 [1]. And with the characteristic slow progression for some tumors and recurrence after treatment in many prostate cancer patients [2], the balance between overaggressive and unneeded treatment coupled with the ability to identify cancers predisposed to aggressive phenotypes remains a challenge. For decades, Prostate Specific Antigen (PSA) had served as the *de facto* standard biomarker for prostate cancer detection. However, it is common knowledge that even in the absence of elevated PSA values, prostate cancer can be present [3]—not uncommonly resulting in false negatives. Wide variation in physiologically normal PSA values, as well as its inconvenient modulation from several sources is a great source of concern for PSA’s clinical utility. In 2011, amid much controversy and intense discussions on a national and international platforms, the U.S. Preventative Services Task Force concluded that “the potential benefit does not outweigh the harms,” with regard to the use of PSA screening as a means to prevent prostate cancer specific mortalities in men [4]. In an era where the most utilized diagnostic marker for prostate cancer has attracted such controversy among many and molecular landscapes and circulating tumor cells of prostate cancer patients are effectively profiled, identification of detection methods with refined sensitivity and precise specificity proceeds at a promising rhythm.

### 2.1. *TMPRSS2:ETS* Gene Fusions

Since their identification in 2005 by the pioneering studies of Arul Chinnayan [5], the *TMPRSS2:ETS* gene fusions have been the focus of intense investigations, as well as a point of contention with regards to its role in prostate cancer as an indicator for the aggressive phenotype. The transmembrane serine protease *TMPRSS2* is androgen regulated, and in response to ligand exposure, fuses with members of the *ETS* family of transcriptional activators [6]. *ERG* is the most commonly fused member of the *ETS* family, with a *TMPRSS2:ETS* fusion occurring in approximately 46% of prostate cancers, in a study of needle biopsies in patients with prostatic malignancy [7]. An exact consensus with regards to the prevalence of these gene fusions in prostate cancer has not been reached due to different detection methods, varying sample sizes, as well as heterogeneity of fusions within the tumor cells. Interestingly, much rigorous debate has surrounded the validity of this lone marker in determining prostate cancer recurrence and mortality, and recent literature calls for a reevaluation of using *TMPRSS2:ETS* fusions alone as prognostic indicators [8]. The important regulatory role of androgens on the *TMPRSS2:ETS* fusion via interaction with the androgen receptor (AR) enables a new promise for a potential role of the gene fusions in predicting the emergence of castration resistant prostate cancer (CRPC). While primary prostate cancer has several treatment options ranging from “active surveillance” to radical prostatectomy, all showing similar and extraordinarily high patient survival rates—with 5 year survival approaching 100% [1], those men who develop CRPC have a poor prognosis and are likely to die as a result of metastatic disease [9]. Androgen deprivation therapy (ADT) is temporarily effective as a treatment strategy to impair prostate cancer by blocking circulating testosterone levels, either by medical or surgical castration. Unfortunately, eventual progression to the castration resistant phenotype is inevitable Sternberg [10].

Specifically relevant to the *TMPRSS2:ETS* fusions and the AR is the transitory dip in expression immediately after androgen deprivation therapy [11]. However, *TMPRSS2:ETS* expression rises once again to levels seen before ADT [11] as primary tumors develop into CRPC. One may argue in favor of future applications to more accurately predict the appearance of the CRPC phenotype by comparing a patient’s baseline and subsequent expression levels of *TMPRSS2:ETS* fusions. The potential benefit to the patient and physician alike would be two-fold: firstly, serving as a more accurate indicator of prognosis; secondly, allowing the physician to better time the introduction of second-line chemotherapeutic agents.

### 2.2. The Androgen Receptor/Transcription Factor-Derived Molecular Signature

The primary target of ADT, the androgen receptor (AR) transcriptionally regulates a series of growth response genes, the intricacies of which have challenged not only their therapeutic exploitation towards the development of effective treatment strategies in CRPC, but also the identification of biomarkers better predicting tumor aggressiveness and ultimate lethality associated with advanced disease. Recent elegant studies by Heemers and colleagues documented the role of the AR in regulation of effector genes along with the transcription factor Serum Response Factor in a 158 gene signature. The resultant androgen dependent gene signature indeed directly correlated with the presence of aggressive disease, poor outcome, biochemical recurrence, as well as demonstrating the ability to accurately distinguish malignant from benign prostate tissue [12]. Similarly striking results were noted by Sharma *et al.* after exploring a 16 gene signature in CRPC, further highlighting the vital role of persistent AR signaling in CRPC [13].

### 2.3. PTEN Loss

The loss of the Phosphatase and Tensin Homolog (*PTEN*) is another genetic aberration involved in prostate cancer, as well as many other types of cancer. *PTEN* acts as a tumor suppressor by encoding for a phosphatase protein product which is involved in cell cycle regulation [14]. Commonly found with a *TMPRSS2:ERG* fusion, the concurrent loss of *PTEN* contributes to a poor prognosis in cancer in the presence of an existing *TMPRSS2:ERG* fusion [15] with an increased risk of recurrence [16]. Although the functional dependence on gene fusions is not clear, *PTEN* has been shown to directly influence tumor progression and early recurrence in both fusion positive and fusion negative tumors [17]. Interestingly, PTEN loss has already shown its utility in an experimental panel of molecular signatures. This four gene panel including *PTEN*, *SMAD4*, *Cyclin D1*, and *SPP1* significantly outperformed Gleason scoring using data from the Physicians’ Health Survey cohort in predicting lethal metastasis, and was further improved when combined with Gleason scoring [18].

### 2.4. Spink 1

Markers for detection and progression of prostate cancer do not completely hinge upon the presence of a gene fusion product. Serine protease inhibitor Kazal type 1 (Spink 1), has been shown to be present in a subtype of prostatic tumors not containing a *TMPRSS2:ETS* fusion [19]. The expression of Spink1 in prostate tumors has shown promise as an independent predictor of biochemical recurrence after resection; most excitingly, is able to be detected in the urine—making it a less-invasive alternative to sampling previous biomarkers [20]. Unfortunately for future clinical application, Spink1 expression is elevated in the absence of a *TMPRSS2:ETS* fusion in only about 10% of cases [20].

### 2.5. Circulating Tumor Cells (CTCs)

In a novel attempt to further tailor treatment to the individual patient, methods of identifying and capturing malignant cells that arise from primary or metastatic tumor sites have been developed. The cell-capture techniques of “moving targets” are constantly improving and guided towards the detection of CTCs in peripheral blood, becoming extremely valuable in the prognosis of cancer patients—in both disease recurrence and likelihood of metastatic invasion [21]. High-cost, inefficient capturing and relatively low levels of circulating CTCs in serum have challenged the routine clinical application and prognostic value of CTCs. However, when CTCs are detected efficiently, initial studies have been quite striking. Moreno *et al.* found a marked correlation between number of CTCs and mortality in patients with metastatic castration resistant prostate cancer; patients with >5 CTCs per 7.5 mL blood had a median survival of 7 years compared with a median survival of 4 years for those patients having <5 CTCs per 7.5 mL blood [22]. More recently, an exciting study from Memorial Sloan-Kettering Cancer Center examined *TMPRSS2:ERG* gene fusion status in CTCs with outcomes in men with CRPC. Men in this study treated with Abiraterone Acetate who demonstrated post therapeutic levels of <5 CTCs per 7.5 mL blood benefitted from a median survival of 72 weeks longer than their cohorts with post therapeutic levels of >5 CTCs per 7.5 mL [23].

### 2.6. The Clustering Value of MicroRNAs

**Figure 1:**
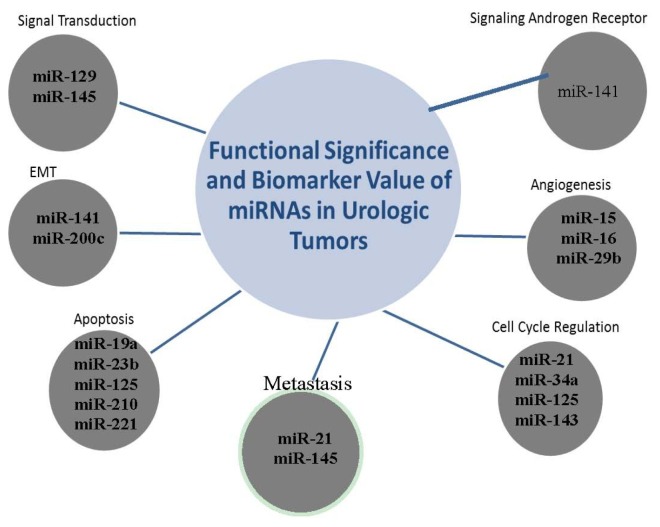
Select miRNAs with potential diagnostic and prognostic utility in urologic tumors, along with selected functions in tumorigenesis/progression of disease. Each miRNA is differentially expressed in malignant states, and contributes to oncogenesis (oncogene activation) or defective tumor suppressor function [27,28].

MicroRNAs (miRNAs*)* represent a unique class of molecules recently discovered and classified as small non-coding RNAs that regulate gene expression at the post-transcriptional level in eukaryotic cells. Since their discovery, miRNAs have emerged as attractive and “sophisticated in their cell-uncoded behavior” molecular species that may have therapeutic targeting value as well as diagnostic potential in tumors. These short (usually ~22 nt) sequences are non-coding segments of RNA regulating approximately 60% of human genes [24]; miRNAs have profound effects on vital cellular functions—including key biological processes involved in tumorogenic transformation and tumor progression to metastasis such as migration, proliferation, and apoptosis [25]. The cancer phenotype can be equally promoted by miRNAs acting excessively on tumor suppressor genes as well as the decreased regulation of proto-oncogenes by miRNAs. Cancer-specific miRNA fingerprints have been identified in many types of cancers, including urologic tumors. Compelling evidence supports a pivotal role for miRNAs in the regulation of cancer stem-like properties of prostate tumors [26] with potential significance in the development of a special platform for molecular therapeutics impairing early events in tumorigenesis, despite the complexity of these intriguing molecules (Figure 1). Moreover certain miRNAs, for example miR32 and miR-145a, are upregulated by androgens in prostate cancer.

### 2.7. miR-141

Although miRNAs might be considered still at their infancy, the emerging scenario of their prominent role in determining future therapeutic strategies and translational outcomes is exciting and rich with promise. Despite the relatively small size of miRNA species, many are robustly expressed in sera of patients with prostate cancer; a study by Brase *et al.* noted that miR-141 served as a potent diagnostic and prognostic indicator in a subset of prostate cancer patient’s sera [29]. Equally important in miR-141’s value as a potential component of a molecular signature panel involves its specificity for malignancies of the prostate, and even more precisely—the presence of aggressive prostate cancer [30]. A schematic summary of several miRNAs found to have specific roles in urologic tumors with potential utility in molecular signature panels is illustrated on Figure 1.

In 2010 and 2011, four new drugs achieved FDA approval targeting metastatic CRPC, bringing much promise in implementation of clever drug design and optimization: Abiraterone Acetate, an androgen biosynthesis inhibitor; cabazitaxel, an inhibitor of mitosis via actions on microtubules; denosumab, targeting bone remodeling processes; sipuleucel-T, an immune system “boosting” vaccine [31]. Moving forward, adaptations in the treatment of prostate cancer toward a systematic, individualized treatment plan based on specific genetic events remains crucial. Further studies elucidating greater understanding of molecular mechanisms functionally linking gene expression profiles to critical processes orchestrating tumor cell behavior are required to further refine treatment options and enhance therapeutic efficacy in patients with CRPC.

## 3. Bladder Cancer

Bladder cancer is the second most common malignancy of the genitourinary tract, and will account for an estimated 15,210 deaths in the United States in 2013 [1]. Five-year relative survival rates remain below 80% for patients diagnosed with bladder cancer [1], and decreasing with more aggressive stages at detection. Bladder tumors and their characteristically high risk of recurrence present an immense challenge in the clinical management of patients and therapeutic outcomes. Significantly enough, due to high recurrence rates and surveillance requiring cystoscopy, bladder cancer is the most expensive cancer to treat in the United States per case—with over half of its cost resulting from surveillance [32]. Growing evidence suggests the ability to predict those tumors with inherently greater risks of recurrence, as well as tumors likely to remain superficial or to invade the bladder wall [33].

### 3.1. Methylation Elevates Genes to Biomarker Status

Exploitation of DNA methylation status in urine specimens of bladder cancer patients is a powerful methodology toward the prediction of tumor presence and aggressiveness. In tumor cells, alterations in DNA methylation of cytosine residues in the form of both hypermethylation and hypomethylation can promote the cancer phenotype. Aberrant hypermethylation of CpG islands of DNA promoter regions silences genes; common sites of hypermethylation in bladder cancer occur at the *WT1*, *BRCA1*, and *RARB* genes [34]. Proteins encoded by these genes serve critical functions in regulating cell differentiation and proliferation, loss of regulation due to methylation of promoter regions has serious deleterious effects [34]—contributing to uncontrolled growth. Hypomethylation, on the other hand, frequently occurs in all regions except CpG islands of promoter regions, which induces genomic instability and gene-specific hypomethylation also promoting carcinogenesis [31,32]. Currently, the gold standard for noninvasive urinary diagnosis of bladder cancer is by urinary cytology—performed by a pathologist or cytologist who categorizes cells in urine as normal, atypical/intermediate, suspicious, or malignant. DNA methylation markers allow for total objectivity as opposed to urinary cytology, and were shown to greatly improve sensitivity when compared with traditional diagnostic strategies [35]. Urine samples are analyzed by two major techniques—methylation sensitive PCR (MSP) and real-time PCR. MSP quantifies the number of methylated/nonmethylated cytosine residues in comparison with reference standards [36]. Methylation markers are more frequently analyzed by real-time PCR due to its low requirement for DNA compared to MSP [37].

## 4. Gene Mutations Shaping Marker Profiles: Really?

### 4.1. *p53*

Commonly referred to as the “guardian of the genome,” *p53* is a significant regulator of the cell cycle and functions as a tumor suppressor protein. The protein product of *p53* tumor suppressor gene, functions by directly binding DNA and repressing transcription of genes that promote growth and invasion; in a malignant state, mutated *p53* is unable to bind DNA, resulting in loss of *p53*’s vital tumor suppressor function [38]. Mutations in the *p53* gene are the most commonly occurring genetic alterations in human cancers, including urologic tumors and often taking a leading causative role in bladder cancer [39]. Clinicopathological evidence indicates that the presence of a mutant *p53* in the bladder urothelium results in a highly aggressive bladder tumor phenotype with a high risk of disease specific mortality [40]. Certain polymorphisms, such as the Pro/Pro genotype at codon 72, have been associated with bladder cancer progression—but fall short in predicting incidence [39]. The presence of a *p53* mutation, leading to abnormal *p53* expression and impacting its function to prevent DNA damage, may potentially serve as an initial physiological stress-factor concerning disease progression; several limitations in its application however as a prognostic marker of bladder cancer must be overcome [41].

### 4.2. *FGF3R*

Mutations in Fibroblast Growth Factor Receptor-3 (*FGF3R*) are more often found in low grade urothelial carcinoma (LGUC) than in high grade urothelial carcinoma (HGUC). A study in 2011 found *FGFR3* mutations in 84% of LGUC, whereas a mutation was present in only 17% of HGUC samples, which did reach statistical significance [42]. The presence of *FGFR3* mutations has generally been found to be associated with low tumor grade, early stage, and low recurrence rate—all leading to a more favorable prognosis [40,43].

### 4.3. Survivin

As a member of the Inhibitor of Apoptosis (IAP) protein family, survivin plays a role in tumor development and progression by inhibiting caspase activity [44]. Although examined in diverse human malignancies, survivin in bladder cancer has been under significant study due to its near universal presence in bladder malignancy, present in 96.3% of samples in a study by Berrada *et al.* [45]. An extensive systematic review by Ku *et al.* showed much greater sensitivity for detection of bladder cancer than did traditional urine cytology [46]. Moreover, survivin has also shown merit as an independent predictor of cancer-specific survival in an elegantly designed clinical study by Shariat and colleagues, revealing that patients with altered survivin expression had a five-year cancer specific survival of 72.9% *versus* 86.2% with normal survivin expression [47].

### 4.4. The *p27* Halt on Cancer Cell Progression

The *p27* gene encodes for a key cell cycle regulatory protein that interacts with cyclin-dependent kinase E towards controlling entry into the cell cycle and ultimately cell proliferation. Over a decade ago, the clinical correlation between loss of p27 protein expression and reduced survival of bladder cancer patients was first documented by Del Pizzo, *et al.* [48]. Consequential to this seminal evidence, additional studies reported that p27 expression inversely correlated with tumor stage and grade in human bladder cancer [33,49]. While p27 was aggressively interrogated as a potential therapeutic for several human malignancies, major culprits of p27 degradation have been elucidated. In normal tissue, p27 functions to inhibit Cdk/cyclin complexes in the nucleus, largely regulating the cell’s journey from G1 to S phase of mitosis. Impaired regulation of the phosphorylation cascade responsible for p27 degradation promotes cell cycle progression, and in turn, uncontrolled cell growth [50]. More recent evidence indicated that mRNA transcribed from the *SKP2* gene was highly elevated, and was once again shown to degrade p27 [33]. Inhibiting the gene products of *SKP2* carries much promise in terms of therapeutic targeting value for the future management of bladder tumors harboring a mutation impacting p27 degradation.

### 4.5. Integrin-Linked Kinase

Integrin-linked kinase (ILK) is a signaling and scaffolding protein that regulates cell survival, proliferation, migration, and angiogenesis [51]. *ILK* gene expression is correlated with tumor invasiveness of bladder cancer in humans, and plays an important role in metastasis [52], predisposing tumors with high ILK expression to a more aggressive phenotype. In addition to showing promise as an indicator of more aggressive, metastatic disease, *ILK* has displayed rather significant potential as a therapeutic target in the treatment of bladder cancer. Knockdown of *ILK* gene expression by siRNAs provides molecular targeting promise as a novel therapeutic strategy towards targeting the multiple and occasionally functionally redundant downstream signaling effectors that play significant roles in navigating cellular processes regulating EMT, inflammation, angiogenesis and anoikis. Diminishing *ILK* expression and the resulting functional consequences on tumorigenesis are mechanistically dictated by the following signaling pathways, inhibition of AKT survival pathway, leading to increased apoptosis of tumor cells; reduction of expression of matrix metalloproteinase-2 (MMP-2) and MMP-9, and upregulation of *nm23-H1*, a potent suppressor gene of metastasis, impairing angiogenesis, vascularity and metastasis [53].

### 4.6. miR-145 and miR-200a

Yun *et al.* recently reported two novel miRNAs (miR-145 and miR-200a) present and stable in urine of bladder cancer patients. In addition to being cell-free and detectable in urine, each of the miRNAs individually demonstrated potential utility in detection and prognostic value in bladder malignancies; miR-145 demonstrated significant correlation with grade, where miR-200a served as an independent predictor of recurrence [54]. Further studies involving exploitation of key mechanistic pathways navigated by these miRNAs will enable validation of their prognostic value in larger numbers of patient cohorts.

## 5. Kidney Cancer

Renal cell carcinoma (RCC) presents a unique challenge for clinicians and investigators alike due to the vast heterogeneity in renal malignancies. As recently as 2012, there were only six FDA approved agents for treatment of RCC, mainly targeting angiogenesis and the mammalian target of rapamycin (mTOR) signaling pathways [55] for the five types of RCC. In 2005, virtually no therapy past surgical intervention and cytokine treatments were available. Even then, cytokine treatment provided only a small chance of long-term benefit, with only approximately 15% of patients showing response to interferon-alpha or IL-2 [56]. Total five-year survival rates in patients with renal malignancies in the years 2001–2007, although significantly improved, remain at only 71% [1]. With 65,150 expected diagnoses of RCC in 2013 and over 13,680 expected deaths as a result [1], marked improvement in the number of treatment options coupled with enhanced efficacy of those options is essential. While there have been significant advances in the development treatment strategies over the last decade targeting malignancies of the kidney, the search for biomarkers with solid prognostic value, specific to renal cell carcinoma has met with considerably less creativity. Central to the understanding of the pathogenesis of RCC is the recognition that an overwhelming majority of molecular markers discovered thus far for RCC are heavily involved in the regulation of angiogenesis, underscoring the role of angiogenesis as critical contributor to the development and progression of RCC, characterized for its extensive vascularization. Neovascularization of tumors is an important process intimately linked to cancer progression and metastasis—as advanced tumor growth is dependent upon excessive angiogenesis in order to access oxygen and nutrients for metabolism. The reactive stroma in the dynamic microenvironment of a developing tumor may also play a primary role in recruiting endothelial cells towards expansive tumor neovascularization and metastatic spread.

### 5.1. *Von Hippel Lindau* (*VHL*) Mutation/Hypoxia Inducing Factor 1-alpha (HIF-1α)

The hypoxia response signaling emerges as a therapeutic target for renal cancer via its causal imposition on tumor vascularity. In response to oxygen changes in the tumor microenvironment, hypoxia inducing factor-α induces angiogenesis [51]. *Von Hippel Lindau* mutations, found in 34%–57% of Clear Cell Renal Cell Carcinoma (ccRCC), cause the dramatically reduction in breakdown of HIF-1α and promote its nuclear translocation, that subsequently transcriptionally activates the master pro-angiogenic gene, *VEGF* [57]. In addition to its angiogenic effects, HIF-1α possesses a multitude of downstream targets, functionally contributing to cell migration and pH modification [58]. Consequently, stabilization of increased HIF-1α levels due to *VHL* gene defects leads to greater probability of metastatic recurrence coupled with lower overall survival rates when compared to renal malignancies not possessing a *VHL* mutation, and has long been established [59]. HIF-1α is involved in upregulating cytokine receptors, specifically CXCR4, that promotes metastasis to specific tissues [60]. The impact of pH modification is discussed below in the context of Carbonic Anhydrase IX function which presents promising value as component of a molecular signature panel in RCC.

### 5.2. Carbonic Anhydrase IX (CAIX)

Fitting the mold as a marker showing relative specificity for clear cell renal carcinoma (ccRCC), CAIX has been identified as an enzyme not present in normal renal tissue, but is present in as many as 97% of ccRCC tumors [61]. Carbonic *Anhydrase IX* is strongly up-regulated in tumors under hypoxic conditions, and is partially responsible for the acidification of the extracellular environment [56]. Acidification of the extracellular matrix promotes tumorigenic transformation, chromosomal rearrangements, extracellular matrix breakdown, and induction of cell growth factor expression [62,63]. CAIX protein expression is strongly correlated with outcome in patients with ccRCC, but its use as an independent marker of prognosis remains suspect [61]. To date, CAIX is undoubtedly one of the most well-defined markers specific to ccRCC; however, its limited validity when used independently calls for the clever application of a molecular signature involving a panel of biomarkers for the purpose of stratifying patients into distinct groups based upon risk of disease progression and mortality.

### 5.3. β-Catenin

The Wnt signaling pathway is a critical regulator of embryonic development, and serves a major role in maintaining cell homeostasis throughout adulthood [64]. Disruption of the *Wnt* pathway ultimately leads to increased levels of a critical transcriptional coactivator β-catenin; accumulation of *β-catenin* leads to transcriptional activation of genes regulating processes involved in malignant development, including proliferation, survival, and differentiation [24]. Accumulation of β-catenin levels is a result of faulty degradation in the cytoplasm by the ubiquitin-protease complex [24]. Significantly enough, mechanistic elucidation of the *Wnt* signaling pathway identified the functional involvement of the *VHL* suppressor gene with β-catenin signaling action in tumor cells [65]. Under normal physiological conditions, VHL suppressor protein possesses an E3-ubiquitin ligase that functions to degrade β-catenin, this loss of function in the VHL protein conferred by mutation of the *VHL* gene affects β-catenin levels in the cell—linking the Wnt signaling pathway to renal malignancy [65].

### 5.4. Hypoxia Induced Factor-2 Alpha (HIF-2α)

HIF-2α is a protein at least partially responsible for regulating the transcription of genes related to erythropoiesis and angiogenesis, glycolysis, and vasodilation [66]. HIF-2α has been implicated in several cancers in addition to RCC, although by engaging a differential hypoxia-mediated pathway than CAIX [67]. Both CAIX and HIF-2α are independent predictors of outcomes in patients with certain malignancies, and their value in combination was shown to be additive [67]. Further studies on the role of hypoxia and its close association with renal cancer hold potentially high clinical significance in the treatment of patients with RCC.

### 5.5. miR-34a

The existing evidence is rather contradicting, as miR-34a has been shown to be elevated in kidney cancer, while it is markedly downregulated in other human malignancies, opening the possibility of miR-34a overexpression as a specific diagnostic marker for renal cell carcinoma [68]. In the same study, it was also established that functional loss of miR-34a markedly inhibited cell proliferation in renal cell carcinoma, providing strong support for the contribution of this molecule to the development of RCC.

## 6. Optimizing Discovery Platforms

Advancements in technology such as high throughput DNA microarrays have revolutionized the efficiency with which researchers are able to catalogue variance in genetic expression between healthy cells and particular types of tumors. However, just as important as gathering vital information is the ability to fully interpret it—which is currently the major obstacle in applying these gene expression profiles for therapeutic use. Downstream effects not yet studied or regulatory steps not yet elucidated or fully understood remain problematic in developing effective treatments tailored to a tumor’s molecular signature. Herein lies another obstacle for clinically significant biomarkers of malignancy in the context of yet-to-be-elucidated molecular pathways—ensuring that “new” markers affecting survival are not purely a product of lead-time bias, but are relevant to carcinogenesis and represent tangible therapeutic targets.

## 7. Conclusions

Global translational research efforts continue, with a focus on the optimization of highly-efficacious therapeutic strategies that benefit patients through the development of treatments fitted to unique genetic and pathophysiological characteristics. Considering the cellular multiplicity (in the context of the microenvironment) and the diversity of the genetic factors contributing to cancer incidence such as the patient genetics and family history, exposure to various diets, lifestyles and cellular heterogeneity that characterizes a multistep, multifactorial disease call for novel treatments tailored to the molecular dynamics of individual tumors—ultimately toward optimization of outcomes in a personalized medicine approach. Identifying the cellular and molecular landscape of individual tumors (EMT, inflammation, growth kinetics, gene methylation status) and targeting the initial molecular events triggering manifestation of tumorigenic growth would allow effective targeted therapeutic strategies and successful clinical outcomes. Collection of gene signatures, including miRNAs panels, hold great promise in enabling investigators to proceed with an accurate diagnosis and physicians to provide patients with appropriately optimized treatment strategies selectively impairing their tumors, eventually leading to cancer prevention and improved quality of life.
